# Airway MMP-12 and DNA methylation in COPD: an integrative approach

**DOI:** 10.1186/s12931-024-03088-3

**Published:** 2025-01-10

**Authors:** Jonas Eriksson Ström, Simon Kebede Merid, Robert Linder, Jamshid Pourazar, Anne Lindberg, Erik Melén, Annelie F. Behndig

**Affiliations:** 1 Department of Public Health and Clinical Medicine, Section of Medicine, Umeå University, 901 87 Umeå, Sweden; 2 Department of Clinical Sciences and Education, Karolinska Institutet, Stockholm, Sweden; 3 Sachs Children’s Hospital, Stockholm, Sweden

**Keywords:** Chronic obstructive pulmonary disease (COPD), DNA methylation, Matrix metalloproteinases (MMPs), Extracellular matrix remodelling, Multiomics, Bronchoscopy

## Abstract

**Background:**

In COPD, the balance between matrix metalloproteinases (MMPs) and their natural inhibitors [tissue inhibitors of metalloproteinases (TIMPs)] is shifted towards excessive degradation, reflected in bronchoalveolar lavage (BAL) as increased MMP concentrations. Because of their critical role in lung homeostasis, MMP activity is tightly regulated, but to what extent this regulation occurs through epigenetic mechanisms remains unknown.

**Methods:**

To explore the interplay between MMPs, TIMPs, and DNA methylation (DNAm) we (1) analysed MMP-9, -12, and TIMP-1 concentrations in BAL fluid, and profiled DNAm in BAL cells from 18 COPD and 30 control subjects, (2) estimated protein–COPD relationships using multivariable regression, (3) identified protein quantitative trait methylation loci (pQTMs) with COPD as a potential modifier in a separate interaction model, and (4) integrated significant interactions with a previous COPD GWAS meta-analysis.

**Results:**

COPD was associated with higher levels of BAL MMP-12 (p = 0.016) but not with MMP-9 or TIMP-1. Further examination of MMP-12 identified association with DNAm at 34 loci (pQTMs), with *TGFBR2* (p = 2.25 × 10^–10^) and *THBS4* (p = 1.11 × 10^–9^) among the top ten pQTM genes. The interaction model identified 66 sites where the DNAm–MMP-12 association was significantly different in COPD compared to controls. Of these, one was colocalized with SNPs previously associated with COPD.

**Conclusions:**

Our findings indicate that airway MMP-12 may partially be regulated by epigenetic mechanisms and that this regulation is disrupted in COPD. Furthermore, integration with COPD GWAS data suggests that this dysregulation is influenced by a combination of environmental factors, disease processes, and genetics, with the latter potentially playing a lesser role.

**Supplementary Information:**

The online version contains supplementary material available at 10.1186/s12931-024-03088-3.

## Background

The extracellular matrix (ECM) of the lungs is made up of proteins and glycoproteins such as elastin, different types of collagens, and fibronectin. Besides providing structural support for cells and vessels, the ECM also acts as a storage space for signalling molecules [1].

Matrix metalloproteinases (MMPs) form a significant family of proteolytic enzymes capable of degrading ECM components, while also modulating cell-to-cell signalling, angiogenesis, and inflammation. In the lungs, alveolar macrophages and pulmonary fibroblasts are the primary sources of MMPs, with additional synthesis by neutrophils, lymphocytes, and epithelial cells [2]. Most MMPs are secreted as inactive precursors and activated extracellularly by enzymes such as serine proteinases, e.g., neutrophil elastase, or other already activated MMPs.

### Regulation of MMPs

To prevent uncontrolled degradation of the ECM, the activity of MMPs is regulated at multiple levels—e.g., expression, secretion, extracellular activation, and inhibition of activation. The latter function is carried out by a group of proteins called ‘tissue inhibitors of metalloproteinases’ (TIMPs), most of which are broad-spectrum inhibitors that can impede the activity of all MMPs, although some display more selective affinities [3]. Plasma protein α2-macroglobulin is another major inhibitor of MMP activity but, unlike TIMPs, α2-macroglobulin is not produced locally in the airways and is thus present there only in very low concentrations.

### COPD, DNA methylation, and MMP regulation

In COPD, the proteolytic balance in the lungs is tipped towards aberrant degradation, as reflected in bronchoalveolar lavage (BAL) by increased concentrations of multiple MMPs, including MMP-9 and MMP-12. Although epigenetic modifications such as DNA methylation (DNAm) are implicated in COPD pathogenesis [4], their specific role in ECM remodelling and proteolytic regulation has not been examined. Potential insights into how DNAm influences these processes may be gleaned from studies of proteolytic imbalances in other inflammatory conditions. In osteoarthritis, for example, increased production of MMPs has been linked to demethylation in their gene promoter regions, which is thought to lead to enhanced synthesis and subsequent cartilage degradation [5]. Changes in chondrocyte DNAm patterns might also contribute to the chronicity of the disease since methylation states are passed on during cell division. Similarly, in diabetic retinopathy, the increased expression of MMP-9 in the retina and its capillaries seems to be maintained through changes in the methylome [6].

### MMP-9, MMP-12, and TIMP-1

MMP-9 and MMP-12 share several characteristics beyond their increased levels in COPD airways. Both can break down elastin and are subject to inhibition by TIMP-1, and both have been proposed as potential biomarkers of COPD and as suitable targets for pharmaceutical interventions [7]. Key differences include their (i) cellular origins—MMP-12 is primarily synthesized by macrophages while MMP-9 is produced in significant amounts by a variety of cell types; (ii) substrate specificity—MMP-12 has a pronounced affinity for elastin, while MMP-9 can also degrade some collagens and gelatin; and (iii) roles in COPD pathogenesis—MMP-12 is prominently implicated in emphysema formation, while both MMPs participate in the regulation of inflammation. TIMP-1 is highly expressed in the lungs and is thought to play a crucial role in ECM homeostasis through inhibition of excessive MMP activity.

We have previously reported a strong association between COPD and DNAm patterns in BAL cells [8]. Many of the implicated pathways were linked to ECM remodelling and MMPs, including transforming growth factor beta (TGF-β) signalling, cell adhesion, and tissue remodelling. Continuing from these findings, the present study aims to explore the interplay between MMPs, their natural inhibitors, and DNAm in the context of COPD. Some of the results have been previously reported in the form of an abstract [9].

## Methods

**Fig. 1:**
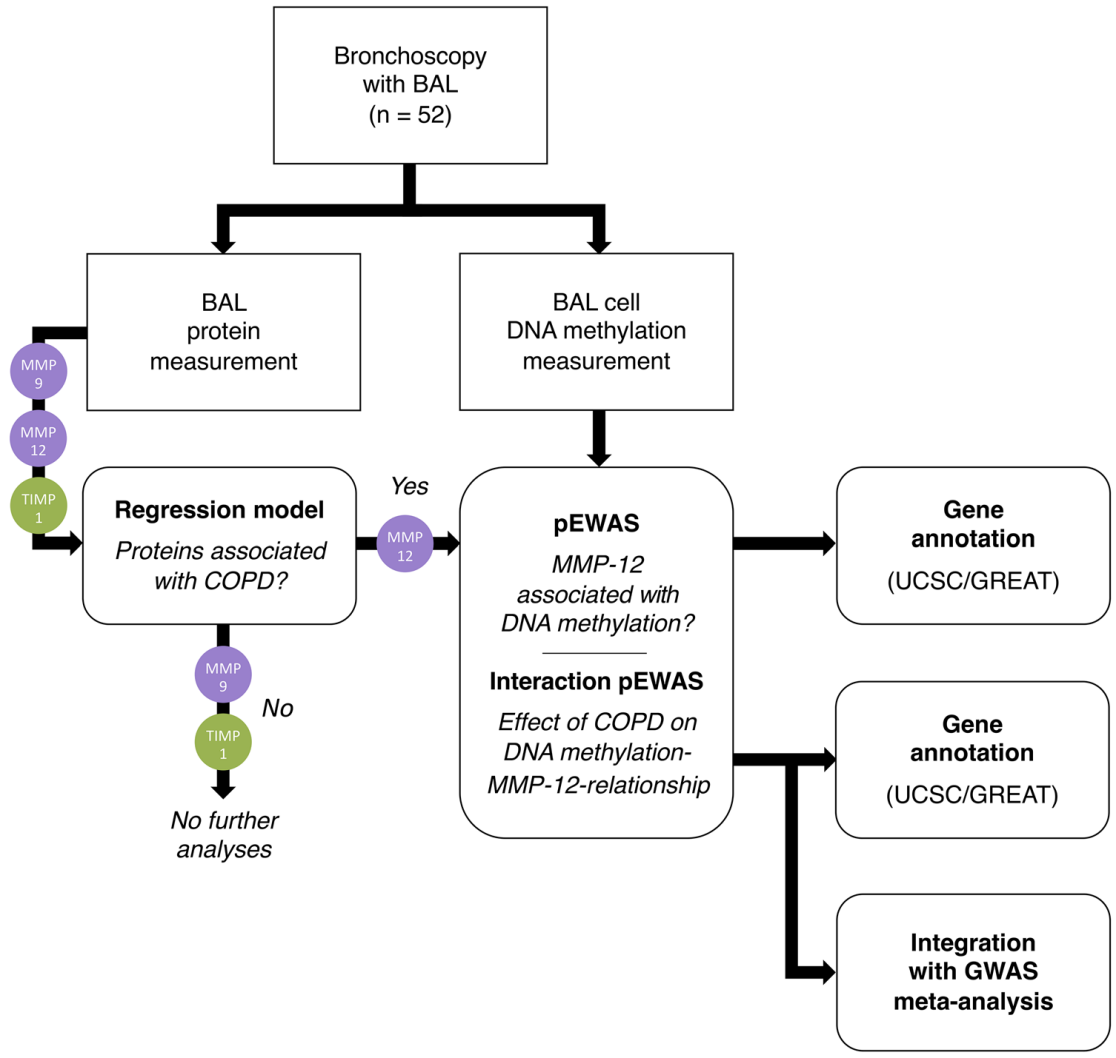
Study overview. DNA methylation in BAL cells and concentrations of proteins MMP-9, MMP-12, and TIMP-1 in BAL fluid were measured. The relationship between COPD and protein concentrations was tested using multivariable regression, and proteins exhibiting significant associations were examined further in a protein-epigenome-wide association study (pEWAS). To evaluate the effect of COPD on protein–DNA methylation-relationships, an interaction pEWAS was implemented. All pQTMs were annotated to genes, and interaction pQTMs were tested for colocalization with genetic variation associated with COPD. BAL: bronchoalveolar lavage; MMP: matrix metalloproteinase; TIMP: tissue inhibitor of metalloproteinases; GREAT: Genomic Regions Enrichment of Annotations Tool; GWAS: genome-wide association study; pQTM: protein quantitative trait methylation loci; UCSC: The University of California, Santa Cruz, Genome Browser Database

For an overview of the methods used in the study, see Fig. 1.

### Study subjects

Twenty-two subjects with COPD and thirty controls with normal lung function participated in this cross-sectional study. All were recruited from the longitudinal OLIN (Obstructive Lung Disease in Northern Sweden) COPD study using predetermined criteria, see online supplementary file for details.

### Bronchoscopy and laboratory analysis

All subjects underwent bronchoscopy with BAL. Three COPD subjects were unable to complete the bronchoscopy procedure. For one subject, BAL recovery rate—the percentage of the instilled saline that is successfully retrieved—was too low to enable analyses. BAL concentrations of MMP-9, MMP-12, and TIMP-1 were assayed using commercially available enzyme-linked immunosorbent assay kits. DNAm in BAL cells was measured using Illumina’s 850 K MethylationEPIC BeadChip, see online supplementary file for details.

### Statistical analyses—airway MMPs/TIMP-1 and COPD

Multivariable regression models were used to test associations between COPD and levels of MMPs/TIMP-1 in BAL. Protein concentrations were transformed using rank-based inverse normal transformation. Sex, age, inhaled corticosteroids (ICS) usage, pack-years, and smoking intensity (number of cigarettes smoked/day) were included as covariates in the models. See online supplementary file for details.

### Statistical analyses—DNA methylation and its association with protein levels

A protein-epigenome-wide association study (pEWAS) was performed to assess the association between DNAm β values and protein levels. To conduct this analysis, we utilized robust linear regression (rlm in the MASS R package). Adjustments included COPD status, sex, age (in years), ICS usage, pack-years, smoking intensity, and cell type counts (including macrophages, lymphocytes, neutrophils, and eosinophils). An interaction model was applied to assess the impact of COPD on the relationships between protein levels and DNAm. To address the issue of multiple testing, we implemented the Bonferroni correction (p < 6.74 × 10^−8^).

### Statistical analyses—integration with GWAS

Protein quantitative trait methylation loci (pQTMs) significantly modified by COPD status were compared to the 82 SNPs from a recent GWAS meta-analysis [10]. We examined if interaction pQTMs in the present study were located within a window 500 kb upstream and 500 kb downstream of these SNPs.

## Results

Data on MMP-9, MMP-12, and TIMP-1 levels in BAL and DNAm in BAL cells was available for 18 subjects with COPD and 30 controls (Table 1). There were no significant differences in age or body mass index between patients and controls. As expected, only COPD subjects used inhaled corticosteroids. The group with COPD had a higher proportion of current smokers, a higher number of pack-years, and—in line with previous studies—a lower BAL recovery rate. Among current smokers, smoking intensity was higher in subjects with COPD. Differential cell counts showed that lymphocytes were lower in COPD compared to controls, and as expected, macrophages were the predominant cell type.

**Table 1:** Study subjects

	COPD (n = 18)	Controls (n = 30)	*p* value
Female:male	4:14	12:18	NS
Age, y	63 (62–72)	65 (63–72)	NS
BMI, kg/m^2^	26.2 (23.7–29.0)	26.1 (24.7–28.4)	NS
Current smokers:Ex-smokers:Non-smokers	10:8:0	3:12:15	< 0.001
Smoking intensity (among current smokers), cigarettes/day	14 (10–20)	8 (7–10)	0.049
Pack-years	36 (30–40)	5 (0–15)	< 0.001
FEV_1_% predicted	68.3 (44.1–72.3)	105.3 (90.5–118.3)	< 0.001
FEV_1_/VC	0.53 (0.43–0.62)	0.76 (0.73–0.80)	< 0.001
Use of inhaled corticosteroids, yes:no	6:12	0:30	0.002
BAL recovery, %	43.6 (32.3–55.6)	64.7 (56.7–68.3)	< 0.001
BAL macrophages, %	89.5 (86.4–92.8)	85.1 (78.8–91.2)	NS
BAL lymphocytes, %	8.3 (5.2–13.2)	13.8 (8.2–19.2)	0.044
BAL neutrophils, %	1.0 (0.4–1.8)	0.8 (0.4–1.2)	NS
BAL eosinophils, %	0.3 (0.0–0.6)	0.2 (0.0–0.4)	NS
BAL mast cells, %	0.2 (0.0–0.5)	0.1 (0.0–0.2)	NS

Values are given as median and interquartile range unless indicated differently. Statistical comparisons: for ratios, the Fisher exact/Fischer-Freeman-Halton test was used, and for other comparisons, the Mann–Whitney U test; a *p* value of less than 0.05 was considered to indicate significance. Pack-years were calculated as (average number of reported cigarettes smoked per day/20) * the years of smoking

BMI: body mass index; COPD: chronic obstructive pulmonary disease; FEV_1_: forced expiratory volume in 1 s; NS: not significant; VC: vital capacity (defined as the highest value of forced and slow vital capacity)

### MMPs/TIMP-1 and COPD

**Fig. 2:**
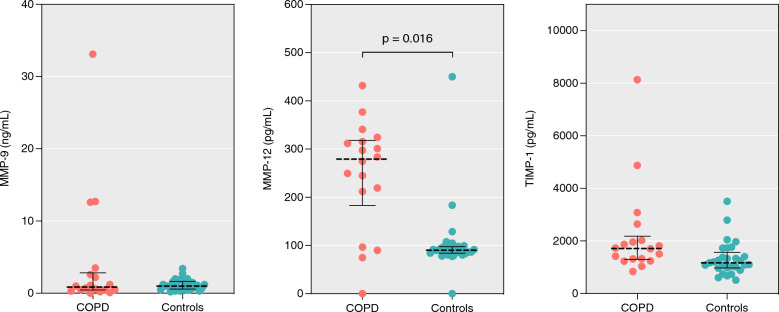
Concentrations of MMP-9, -12, and TIMP-1 in bronchoalveolar lavage. Dotted line indicates median, bars the interquartile range. MMP: Matrix metalloproteinase. TIMP: tissue inhibitor of metalloproteinases. COPD: chronic obstructive pulmonary disease

COPD was associated with higher levels of MMP-12 in BAL (β = 0.445, p = 0.016) but no significant associations were found between COPD and TIMP-1 or MMP-9 (see Fig. 2). TIMP-1, but none of the measured MMPs, was positively associated with smoking intensity (β = 0.530, p = 0.002). See online supplementary file for full results.

### DNA methylation, MMP-12, and COPD

**Fig. 3:**
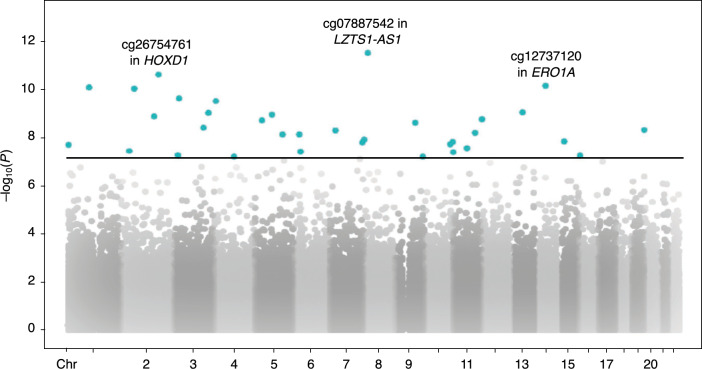
Association between airway MMP-12 and DNA methylation. pEWAS Manhattan plot. Three most significant pQTMs labelled with annotated genes. Black line represents the Bonferroni-corrected threshold for significance. pEWAS: protein-epigenome-wide association study; MMP-12: Matrix metalloproteinase 12; pQTM: protein quantitative trait methylation loci; Chr: chromosome; *HOXD1*: Homeobox D1; *LZTS1-AS1*: LZTS1 Antisense RNA 1; *ERO1A*: Endoplasmic Reticulum Oxidoreductase 1 Alpha

Since MMP-12 displayed a significant relationship with COPD, this protease was examined further. In the first pEWAS, we examined the relationship between DNAm and MMP-12 while adjusting for COPD status to account for disease-specific effects. This analysis identified 34 Bonferroni significant pQTMs (see Fig. 3, and Table 2 and S1). Among the ten pQTMs with the lowest *p* values, cg22581895—annotated to *TGFBR2*—displayed the largest effect size. About half of the pQTMs (18/34) displayed a positive association between methylation and MMP-12.

**Table 2:** The top 10 pQTMs, indicating associations between MMP-12 and DNA methylation, ranked by *p* value

CpG ID	Chr	Genomic coordinate	Annotated to gene	Coefficient	*p* value
cg07887542	8	20142604	LZTS1-AS1^a^	8.56	3.01 × 10^–12^
cg26754761	2	177040938	HOXD1^b^	4.82	2.28 × 10^–11^
cg12737120	14	53113169	ERO1A^a^	22.5	6.90 × 10^–11^
cg19793753	1	100608297	TRMT13^a^	16.5	7.96 × 10^–11^
cg10669976	2	62667417	TMEM17^b^	− 19.0	8.75 × 10^–11^
cg22581895	3	30352425	TGFBR2^b^	22.8	2.25 × 10^–10^
cg05568532	4	7136338	SORCS2^b^	14.1	2.95 × 10^–10^
cg13193392	13	60055857	DIAPH3^b^	− 20.4	8.80 × 10^–10^
cg08777395	3	167682775	GOLIM4^b^	− 21.8	9.15 × 10^–10^
cg00795341	5	79330929	THBS4^a^	13.6	1.11 × 10^–09^

Adjusted for COPD, sex, age, inhaled corticosteroid usage, cell type, pack-years, and smoking intensity

pQTM: protein quantitative trait methylation loci; MMP-12: Matrix metalloproteinase 12; *LZTS1-AS1*: LZTS1 Antisense RNA 1; *HOXD1*: Homeobox D1; *ERO1A*: Endoplasmic Reticulum Oxidoreductase 1 Alpha; *TRMT13*: TRNA Methyltransferase 13 Homolog; *TMEM17*: Transmembrane Protein 17; *TGFBR2*: Transforming Growth Factor Beta Receptor 2; *SORCS2*: Sortilin-Related VPS10 Domain Containing Receptor 2; *DIAPH3*: Diaphanous Related Formin 3; *GOLIM4*: Golgi Integral Membrane Protein 4; *THBS4*: Thrombospondin 4

^a^Illumina annotation is based on the University of California, Santa Cruz (UCSC) database, using the hg19/GRCh37 genome assembly

^b^Nearest gene annotations are reported by the Genomic Regions Enrichment of Annotations Tool (GREAT), version 4.0.4, also based on the UCSC hg19/GRCh37 genome assembly

**Fig. 4:**
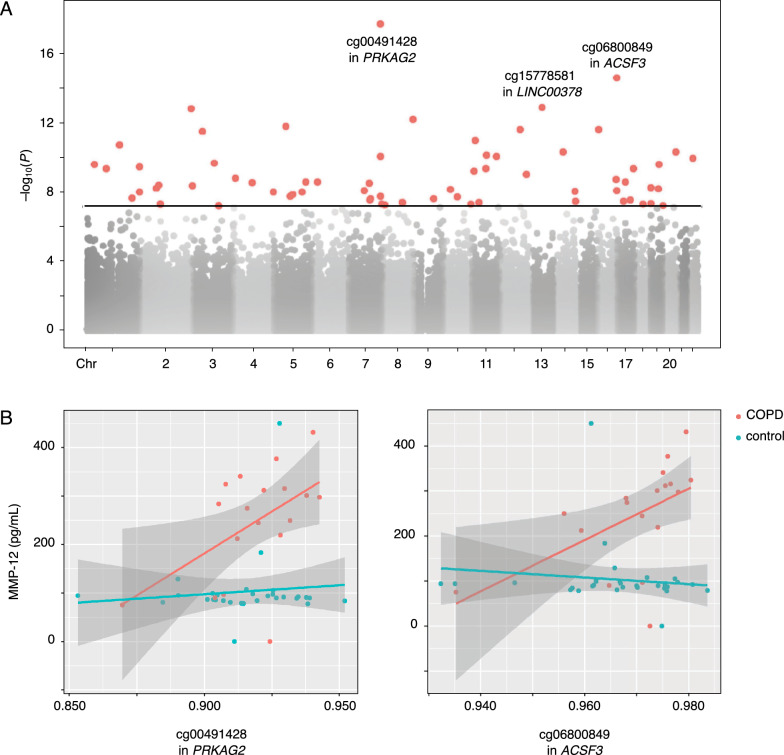
The effect of COPD on the MMP-12–DNA methylation relationship. **A** Interaction pEWAS Manhattan plot indicating interactions between COPD and DNA methylation associated with MMP-12. Three most significant interaction pQTMs labelled with annotated genes. Black line represents the Bonferroni-corrected threshold for significance. **B** Selected interaction pQTMs. X-axis: DNA methylation, y-axis: MMP-12 concentrations. pEWAS: protein-epigenome-wide association study; MMP-12: Matrix metalloproteinase 12; pQTM: protein quantitative trait methylation loci; Chr: chromosome; COPD: chronic obstructive pulmonary disease; *PRKAG2*: Protein Kinase AMP-Activated Non-Catalytic Subunit Gamma 2. *ACSF3*: Acyl-CoA Synthetase Family Member 3; *LINC00378*: Long Intergenic Non-Protein Coding RNA 378

In the interaction model, we added COPD as a potential modifier of the relationship between DNAm and MMP-12 levels. This analysis identified 66 Bonferroni significant interaction pQTMs of which about two-thirds (41/66) were positive associations (see Figs. 4 and S1, and Table 3). None of the interaction pQTMs were within ± 500 kb of the MMP-12 transcription start site (see Table S5). Since interaction analyses can be sensitive to outliers, we conducted a sensitivity analysis excluding DNA methylation outliers. This did not significantly affect the results (see online supplementary file for details).

**Table 3:** The top 10 interaction pQTMs, indicating interactions between COPD and DNA methylation associated with MMP-12, ranked by interaction *p* value

CpG ID	Chr	Genomic coordinate	Annotated to gene	Interaction coefficient	Interaction *p* value
cg00491428	7	151553868	PRKAG2^a^	46.9	1.93 × 10^–18^
cg06800849	16	89180587	ACSF3^a^	72.5	2.33 × 10^–15^
cg15778581	13	61259372	LINC00378^a^	29.5	1.27 × 10^–13^
cg15854830	2	241762029	KIF1A^b^	32.5	1.46 × 10^–13^
cg00635382	8	143824558	SLURP1^a^	132	6.16 × 10^–13^
cg12756648	5	55617397	IL6ST^b^	− 35.0	1.54 × 10^–12^
cg07241613	12	93690962	LOC643339^a^	9.97	2.33 × 10^–12^
cg22630180	16	3897611	CREBBP^a^	− 115	2.33 × 10^–12^
cg20540694	3	50467313	CACNA2D2^a^	23.4	2.89 × 10^–12^
cg03174644	11	15465937	INSC^b^	49.0	1.05 × 10^–11^

Adjusted for sex, age, inhaled corticosteroid usage, cell type, pack-years, and smoking intensity

pQTM: protein quantitative trait methylation loci; MMP-12: Matrix metalloproteinase 12; *PRKAG2*: Protein Kinase AMP-Activated Non-Catalytic Subunit Gamma 2; *ACSF3*: Acyl-CoA Synthetase Family Member 3; *LINC00378*: Long Intergenic Non-Protein Coding RNA 378; *KIF1A*: Kinesin Family Member 1A; *SLURP1*: Secreted LY6/PLAUR Domain Containing 1; *IL6ST*: Interleukin 6 Cytokine Family Signal Transducer; *LOC643339*: Uncharacterized LOC643339; *CREBBP*: CREB Binding Protein; *CACNA2D2*: Calcium Voltage-Gated Channel Auxiliary Subunit Alpha2delta 2; *INSC*: INSC spindle orientation adaptor protein

^a^Illumina annotation is based on the University of California, Santa Cruz (UCSC) database, using the hg19/GRCh37 genome assembly

^b^Nearest gene annotations are reported by the Genomic Regions Enrichment of Annotations Tool (GREAT), version 4.0.4, also based on the UCSC hg19/GRCh37 genome assembly

### Integration with GWAS

To examine the colocalization of epigenetic and genetic variation, interaction pQTMs in the present study were mapped to the 82 SNPs associated with COPD in a large-scale GWAS meta-analysis [10]. We found that 1/66 significant interaction pQTMs were within a ± 500-kb window of COPD-associated SNPs (cg10645426 in *HSPA4*; detailed results in Table S4).

## Discussion

### Increased MMP-12 in COPD

To determine the proteolytic activity in COPD airways, we measured concentrations of MMP-9, MMP-12, and TIMP-1 in BAL fluid from COPD subjects and controls. In line with many previous studies, we found that MMP-12 was significantly increased in COPD, but for MMP-9 and TIMP-1, no such associations were found.

Several mechanisms might contribute to increased levels of MMP-12 in COPD airways. For example, microbial colonization and impaired phagocytosis can lead to persistent activation of immune cells and production of pro-inflammatory cytokines. These cytokines, in particular TNF-α and IL-1β [11, 12], stimulate macrophages and other cell types to produce MMP-12. Interestingly, based on the same study population as the current, we have previously reported that NK cells, capable of producing both TNF-α and IL-1β, were increased in COPD subjects [13]. Dysfunctional mitochondria are another well-described feature of COPD [14] and one that can further contribute to increased airway MMP-12. Leaky mitochondrial membranes generate reactive oxygen species that activate the NF-κB pathway, contributing to increased expression of MMP-12 [15].

In addition to these effects of chronic disease processes, in current smokers with COPD, the secretion of MMP-12 may also be influenced by acute effects of smoking. For example, tobacco smoke has been shown to cause leakage of serum containing plasminogen and prothrombin into the alveoli. After being activated, these proteins can bind to alveolar macrophage PAR-1 receptors [16], which leads to secretion and activation of MMP-12. Other mechanisms include upregulation of granulocyte–macrophage colony-stimulating factor (GM-CSF) and tachykinins [1], e.g., substance P and neurokinin, both of which have been suggested to increase secretion of macrophage MMP-12. In our study, smoking intensity was however not a significant predictor of MMP-12 concentrations.

### The chicken or the egg?

The above-described mechanisms relate to MMP-12 in manifest COPD. However, increasing evidence of early-life events and genetics as major factors for the development of COPD raises the question of what comes first: manifest COPD or increased activity of MMP-12 in the airways? (Or even a causal relationship in both directions?).

It is widely recognized that in utero and early life tobacco smoke exposure leads to long-term adverse effects on lung function and increased risk for COPD. In mice models, in utero secondhand smoke exposure causes upregulation of *MMP-12* at gene and protein levels and leads to significantly enlarged airspaces in the offspring [17]. MMPs are vital for normal lung development in mice as well as humans, with MMP-12 being expressed in the later stages of development [18]. During childhood, tobacco smoke exposure, lower respiratory tract infections, and asthma are risk factors for the later development of COPD [19]. In a longitudinal study from Tasmania, 75% of individuals who developed COPD in their 50 s had exhibited impaired lung function already during childhood [20].

As for genetics, it has been found in several cohorts that the minor allele of an SNP in the promoter of the *MMP-12* gene (substitution A-to-G at position − 82; rs2276109) is associated both with a reduced risk of developing COPD and with a beneficial effect on lung function in children with asthma [21]. This functional polymorphism impairs AP-1 transcription factor binding, resulting in a lower expression of MMP-12. In another study, haplotypes of two SNPs in *MMP-12* (rs652438 and rs2276109) were associated with severe to very severe COPD [22].

Based on our findings, the potential mechanisms described above, as well as the large body of evidence from previous studies supporting a major role for MMP-12 in COPD pathogenesis, we decided to investigate this matrix metalloprotease further in a pEWAS.

### Association between MMP-12 and DNA methylation

To the best of our knowledge, this was the first time the role of DNAm in airway MMP-12 regulation was explored. Our pEWAS identified 34 loci where levels of DNAm in BAL cells were significantly associated with concentrations of MMP-12 in BAL. This model specifically estimated the relationship in a non-COPD context, highlighting shared regulatory mechanisms of MMP-12 across healthy and diseased states. Among genes annotated to the 10 pQTMs with the lowest *p* values, two are well-known to be associated with MMP-12 and ECM remodelling: *TGFBR2* (Transforming Growth Factor Beta Receptor 2) and *THBS4* (Thrombospondin 4)*.*

*TGFBR2* encodes a transmembrane protein acting as a receptor for the TGF-β signalling pathway, which can stimulate the expression of ECM proteins, the activity of MMPs in general, and MMP-12 in particular [11]. TGFBR2 is also implicated in interactions with other molecules associated with ECM remodelling, such as integrins, focal adhesion kinases, and thrombospondins.

THBS4 (also known as TSP-4) is an ECM protein and a member of the thrombospondin family whose expression is closely associated with TGF-β signalling [23]. Thrombospondins function to guide tissue remodelling and extracellular matrix synthesis through interactions with other ECM proteins and MMPs. *THBS4* has been associated with interstitial lung disease [24], a group of conditions in which MMP-12 is often increased and plays a pro-fibrotic role [25].

Among the remaining top ten pQTM genes, four have previously been found to interact with MMP-12 or other MMPs in different settings (*HOXD1*, *DIAPH3*, *TMEM17*, and *SORCS2*); three to be involved in ECM interactions (*ERO1A*, *GOLIM4*, and *TMEM17*); while two have no previously known relationship with MMPs or ECM remodelling (*LZTS1 Antisense RNA 1* and *TRMT13*). The biological importance of ECM-related gene regulation is underscored by the fact that six of our top ten pQTM genes are associated with either lung cancer or interstitial lung disease.

### The effect of COPD on the MMP-12—DNA methylation relationship

Next, we sought to examine to what extent changes in DNAm might explain the increased MMP-12 in COPD airways. Here, we employed an interaction model in which COPD was included as a potential modifier. This analysis identified 66 interaction pQTMs, i.e., sites where the relationship between DNAm and MMP-12 levels was different in COPD compared to controls (e.g., slope of, variability around, or intercept of the regression line; see Figs. 4 and S1 for examples). For some interaction pQTMs, both mean methylation and MMP-12 concentrations were elevated in COPD subjects compared to controls. However, even when mean methylation was similar across groups, concentrations of MMP-12 were generally higher in those with COPD. This suggests that both DNAm and other mechanisms may contribute to the upregulation of MMP-12 in COPD airways.

Among the top 10 interaction pQTMs, eight were annotated to protein-coding genes, and all of those have previously been associated with either COPD or smoking (*PRKAG2*, *ACSF3*, *IL6ST*, *CREBBP*, *KIF1A*, *SLURP1*, *CACNA2D2*, and *INSC*), indicating biologically plausible results, and also a potential role for these genes in the dysregulation of MMP-12 in COPD. The remaining two interaction pQTM genes were long non-coding RNAs of which little or no mention can be found in the literature (*LINC00378* and *LOC643339*).

*PRKAG2* (Protein kinase AMP-activated non-catalytic subunit gamma 2) encodes for a sub-unit of the AMP-activated protein kinase (AMPK) enzyme, a central regulator of cellular energy metabolism implicated in COPD in several ways (e.g., compensation for mitochondrial dysfunction, regulation of oxidative stress and smooth muscle contraction). AMPK can be deactivated by MMP-12 proteolytic activity, leading to an increase of TNF-α [26]. Conversely, activation of AMPK seems to suppress the expression of at least some MMPs, although it is not clear if this applies to MMP-12 [27]. At the signalling level, this crosstalk between MMP and AMPK pathways might modulate inflammatory responses in the lung and has been suggested to play a role in emphysema development [28]. Based on machine learning analyses of gene expression, *PRKAG2* has been proposed as a potential therapeutic target in COPD [29], it has also been found to be differentially methylated in several previous studies of the disease [30, 31].

ACSF3 (Acyl-CoA synthetase family member 3) is part of the acyl-CoA synthetase family, involved in fatty acid metabolism, and located exclusively in the mitochondrial matrix in humans [32], although its role in mitochondrial dysfunction in COPD specifically has not been examined. In an epigenetic study exploring the developmental origins of COPD, *ACSF3* was found to be differentially methylated in smoke-exposed foetal lung samples compared to unexposed samples [33]. It has also been identified as a key regulator of COPD in an integrated analysis of lung tissue DNAm and gene expression [34].

### Causal direction

Based on the currently available evidence, the causal direction of the above-described relationships is not fully elucidated, i.e., whether MMP-12 regulates DNAm or the other way around. For at least some MMPs, there is experimental support for the latter hypothesis; inhibition of DNAm has been shown to reduce methylation in MMP gene promoter regions, and this in turn to result in increased transcription and secretion of MMPs [35]. However, the alternative hypothesis, that airway MMP-12 could affect methylation of BAL cell DNA, is also biologically plausible. MMPs can alter cytokine activity in several ways—e.g., via interaction with neutrophils, activation of pro-cytokines through proteolytic cleavage, and ECM remodelling leading to the release of stored cytokines and activation of pro-inflammatory signalling pathways. Experimental evidence indicates that some of these cytokines (IL-1β and TNF-α in particular) can affect the activity of DNA methyltransferases and thus potentially DNAm [36, 37].

### Integration with GWAS

In contrast to the order of nucleobases in the DNA, methylation marks are dynamic—they accumulate over time and can change due to environmental cues. However, at some loci, DNAm is strongly associated with genetic variants, i.e., sites where DNA sequence somehow shapes methylation patterns. This phenomenon has been reported for genetic variants (SNPs) associated with many diseases, including COPD [38].

As genetic determinants are important for COPD pathogenesis, we tested whether the identified epigenetic variation related to both MMP-12 and COPD was colocalized with genetic variants linked to the disease. To do this, we integrated our interaction pQTM results with those from a recent large-scale GWAS of 35,735 cases and 222,076 control subjects [10]. We found that merely 1/66 interaction pQTMs were colocalized with COPD-associated SNPs. This may suggest that factors influencing MMP-12 overexpression in COPD airways are more strongly related to environmental influences and disease processes than to genetic predispositions. However, genetic effects on MMP-12 expression could still manifest through mechanisms not captured by colocalization analysis, such as transcriptional regulation, post-transcriptional modifications, or epigenetic changes in other cell types or tissues.

### Potential mechanisms

The classic mechanism by which DNAm alters gene expression involves promoter methylation, which inhibits the binding of transcription factors and subsequently leads to decreased expression of the target gene. It might thus seem unexpected that *MMP-12* was not in the list of interaction pQTM genes. However, the process from DNA sequence to protein expression is in reality more complex, and there are several potential mechanisms that could explain the effect of COPD and DNAm on airway MMP-12 levels and activity. For example, DNAm could affect (through cis- or trans-effects) the availability and expression of (i) proteinases involved in the extracellular cleavage of MMP-12 prodomains—since MMPs are most often secreted as inactive proenzymes [7]; (ii) transcription factors and/or endogenous small RNAs that affect MMP-12 expression [39]; and/or (iii) enzymes involved in the post-translational machinery, since MMP-12 secretion and activity may be altered by for example phosphorylation [40]. As discussed above, the causal relationship between DNAm and MMP-12 might also be bidirectional, i.e., some interaction pQTMs could represent sites where methylation is affected by MMP-12 and/or other COPD processes and not the other way around.

### Strengths and limitations

Currently, no other dataset that contains measurements of MMPs and DNAm levels in BAL has been published. Consequently, our findings could not undergo validation in a suitable external dataset. While MMP/DNAm datasets based on peripheral blood exist, the marked differences in DNAm patterns and MMP-12 concentrations between compartments make replication in such a dataset inherently challenging to interpret. So, although our analyses indicate that we did capture a relevant signal (e.g., eight out of the top ten pQTM genes were previously associated with MMPs/ECM remodelling, and all top ten protein-coding interaction pQTM genes with COPD/smoking), availability of an external dataset enabling validation would have further reinforced our findings.

Another constraint is the limited number of participants included. While this limitation is common in studies relying on bronchoscopies, a larger study cohort would have been desirable. Furthermore, the ELISA kit used to measure proteins in BAL could not discriminate between active and inactive forms of MMPs.

The absence of genotype data in our cohort limits our ability to directly evaluate cis-meQTLs or explore SNP-methylation-protein interactions within the study population. For example, we were unable to test the potential impact of rs2276109, a SNP shown to affect both COPD risk and MMP-12 expression [21], on DNAm or protein levels. Integration with GWAS meta-analysis data provided an alternative approach to investigating the relationship between genetic and epigenetic variation, enabling us to contextualize our findings within a larger, well-characterized dataset.

One major strength is the well-characterized study population. Statistical adjustments are a powerful tool, but their effectiveness is entirely dependent on the quality of the control variable data. In the current study, all subjects were recruited from the longitudinal OLIN COPD study which provided detailed and reliable information on smoking habits, use of ICS, and other variables included as covariates in the models. Another strength is the use of BAL and not surrogate tissue such as peripheral blood. When trying to understand processes that take place in the lungs (e.g., expression and secretion of airway MMPs, in health and disease), examining target organ tissue is more likely to produce more relevant information than surrogates. Furthermore, integrating several layers of omics data allows for a more complete understanding of disease mechanisms and may provide more robust results, especially when sample size is limited [41].

## Conclusions

Using a hypothesis-driven approach, we show for the first time (i) that airway MMP-12 may partially be regulated through DNA methylation; and (ii) that this regulatory mechanism seems to be disrupted in COPD, with overexpression of MMP-12. Integration with COPD GWAS datasets suggests that this dysregulation is influenced by a combination of environmental factors, disease processes, and genetics, with the latter potentially playing a lesser role. Further investigations are required to determine the causal directions of these associations with greater certitude.

## Supplementary Information

Additional file 1: Additional details on methods and results including Table S1 and Figure S1.

Additional file 2: Full pEWAS, interaction pEWAS, and GWAS integration results in datasheet format (Tables S2–S5).

## Data Availability

The datasets used and/or analysed during the current study are available from the corresponding author on reasonable request.

## Abbreviations

BAL: Bronchoalveolar lavage
BMI: Body mass index
COPD: Chronic obstructive pulmonary disease
DNAm: DNA methylation
ECM: Extracellular matrix
ELISA: Enzyme-linked immunosorbent assay
FEV_1_: Forced expiratory volume in the first second
GREAT: Genomic Regions Enrichment of Annotations Tool
GWAS: Genome-wide association study
ICS: Inhaled corticosteroids
MMP: Matrix metalloproteinase
pEWAS: Protein-epigenome-wide association study
pQTM: Protein quantitative trait methylation loci
SNP: Single nucleotide polymorphism
TGF-β: Transforming growth factor beta
TIMP: Tissue inhibitor of metalloproteinases
UCSC: The University of California, Santa Cruz, Genome Browser Database
VC: Vital capacity
