# Supplementary material for: Airway MMP-12 and DNA methylation in COPD: an integrative approach
Source: Respir Res. 2025 Jan 10;26:10. doi: 10.1186/s12931-024-03088-3 (PMC11724436; doi:10.1186/s12931-024-03088-3)
Supplement: Supplementary file 1 — Additional file 1: Additional details on methods and results including Table S1 and Figure S1. [file 12931_2024_3088_MOESM1_ESM.docx]

SUPPLEMENTARY FILE

**Airway MMP-12 and DNA methylation in COPD: an integrative approach**

Jonas Eriksson Ström^1,*,†^, Simon Kebede Merid^2,*^, Robert Linder^1^, Jamshid Pourazar^1^, Anne Lindberg^1^, Erik Melén^2,3,‡^, and Annelie F. Behndig^1,‡^

^1^ Department of Public Health and Clinical Medicine, Section of Medicine, Umeå University, Umeå, Sweden; ^2^ Department of Clinical Sciences and Education, Karolinska Institutet, Stockholm, Sweden; and ^3^ Sachs Children’s Hospital, Stockholm, Sweden. ^*^ shared first authorship, these authors contributed equally to this work; ^†^ corresponding author; and ^‡^ shared last authorship, these authors contributed equally to this work

**Methods**

*Study subjects*

There were no reports of exacerbations in the four weeks prior to bronchoscopy. Individuals with inflammatory conditions or medications that could potentially influence the study's results, or health conditions that posed contraindications to bronchoscopy were not included in the study cohort. The procedure for participant selection, along with the criteria for inclusion and exclusion, has been documented previously[1]. Informed consent was obtained from all volunteers after verbal and written information. The local Ethics Committee at Umeå University, Sweden, granted approval for the study, which was performed in accordance with the Declaration of Helsinki.

*Bronchoscopy*

Bronchoscopies were carried out by a single medical team operating at two distinct sites: the Division of Respiratory Medicine and Allergy, Department of Medicine, Sunderby Central Hospital of Norrbotten, Luleå, Sweden, and the Division of Respiratory Medicine and Allergy, Department of Medicine, University Hospital, Umeå, Sweden. Topical anaesthesia was achieved using lidocaine. Prophylactic measures included administering 1.0 mg of atropine subcutaneously to the subjects half an hour before the bronchoscopy. In some cases, 4–8 mg of midazolam per so was also given. A flexible video bronchoscope was inserted through the mouth via a mouthpiece with the subject supine. Bronchoalveolar lavage (BAL) involved the introduction of three 60 ml aliquots of sterile sodium chloride solution (0.9% concentration, pH 7.3, warmed to 37°C) into either the middle lobe or the lingula. The lavage fluid was then aspirated back and pooled into a tube placed in iced water. The recovered BAL fluid was immediately transported to the laboratory for analysis. Bronchial wash (2 × 20 ml) and biopsies were also performed but not included in the analyses in the current study.

*Laboratory analyses*

BAL samples were frozen and stored at –80˚C until analysis. All samples from all subjects were analysed in a single batch on the same day. BAL concentrations of MMP-9, MMP-12, and TIMP-1 were assayed using commercially available enzyme-linked immunosorbent assay (ELISA) kits (Duo Set® ELISA Development System, R&D Systems Europe Ltd., United Kingdom), according to the manufacturer’s instructions. Recombinant human MMP-9, MMP-12, and TIMP-1 were used to construct a standard curve (range 39–2,500 pg/mL) for each set of samples assayed. BAL samples were diluted 1:2, and concentrations read from the standard curve were multiplied by the dilution factor.

*DNA methylation*

We assessed epigenome-wide DNA methylation (DNAm) levels using DNA isolated from BAL cells. From each sample, a 500 ng aliquot of DNA was subjected to bisulfite conversion using the EZ-96 DNA Methylation Kit (Zymo Research Corporation, Irvine, CA, USA). Samples were then arranged in a random sequence on 96-well plates for analysis with the MethylationEPIC BeadChip, following the standard protocol provided by the manufacturer (Illumina Inc., San Diego, CA, USA) at the SNP&SEQ Platform at Uppsala University. This chip analyzes 866,836 CpG sites throughout the genome.

Quality control of analysed samples was performed using standardized criteria. Raw methylation intensities were processed using GenomeStudio Software, and the detection *p* value of each CpG site was used as a quality control measure of probe performance. To reduce background noise and adjust for variations across arrays, we utilized the Bioconductor Illumina Minfi and ComBat packages[2]. These tools were also used for peak correction and batch effect mitigation. We estimated DNA methylation at each CpG site as the proportion of intensity of methylated (M) over the sum of methylated (M) and unmethylated (U) probes, β = M/[c + M + U], where c is a constant used to avoid division by zero. Any CpG sites with detection *p* values >.01 in more than 5% of all samples, or those that were not successfully measured in 5% of the samples, were omitted from further analysis. This led to the exclusion of 8,401 probes.

Samples were excluded if the sample call rate was <3. Factors such as colour, staining and extension efficiency, hybridization performance, stripping efficiency after extension, and bisulphite conversion efficiency were assessed using the Enmix R package[3]. Using the Minfi R package, we also generated median intensity plots, graphically representing the distribution of both methylated and unmethylated signal intensities across samples. No samples were excluded by applying these criteria.

All SNP-related probes identified by Zhou and colleagues[4], along with probes with nonoptimal binding (non-mapping or mapping multiple times to either the normal or the bisulphite-converted genome[5]; n = 97,018), and probes on chrX (n = 18,578), were omitted, leading to a total exclusion of 123,997 probes. Additionally, we adopted the DASEN signal correction and normalization method recommended from the wateRmelon package[6]. Batch effects were corrected using ComBat[2].

*Gene annotation*

For target genes, Illumina's annotation based on the University of California (UCSC), Santa Cruz, database (hg19/GRCh37 genome assembly) was used, utilizing the UCSC gene transfer format file corresponding to this genome build. In cases where no target gene was available, Genomic Regions Enrichment of Annotations Tool (GREAT) version 4.0.4 annotation[7] (also based on the UCSC hg19/GRCh37 genome assembly) was instead used to find nearest gene.

*Statistical analysis – Airway MMPs/TIMP-1 and COPD*

Multivariable regressions were calculated to predict BAL levels of proteins (MMP-9, MMP-12, and TIMP-1) based on COPD status, sex, age, ICS usage, pack-years, and smoking intensity (number of cigarettes/day). Protein levels were transformed using rank-based inverse normal transformation (Blom’s proportion estimation formula as implemented in SPSS). For all models, COPD status was coded as 1 = COPD and 0 = non-COPD, sex as 1 = female and 2 = male, age was measured in years, ICS usage was coded as 1 = yes and 0 = no, pack-years was defined as the number of packs of cigarettes smoked per day multiplied by the number of years the subject had smoked, and smoking intensity was measured as the number of cigarettes smoked/day.

**Results**

*MMPs/TIMP-1 and COPD*

For MMP-12, a significant regression equation was found (F(6, 41) = 3.413, p = 0.008), with an R^2^ of 0.333. Participants’ predicted transformed MMP-12 was equal to - 0.111 + 0.313 (COPD status) + 0.040 (sex) + 0.006 (age) -0.050 (ICS usage) + -0.001 (pack-years) + 0.007 (smoking intensity). COPD status was a significant predictor of transformed MMP-12 levels in BAL.

For MMP-9, no significant regression equation could be found (F(6, 41) = 2.023, p < 0.084), with an R^2^ of 0.228. Participants’ predicted transformed MMP-9 was equal to - 0.629 - 0.035 (COPD status) + 0.132 (sex) + 0.013 (age) - 0.214 (ICS usage) + 0.003 (pack-years) + 0.001 (smoking intensity). No significant predictors of transformed MMP-9 levels in BAL were identified.

For TIMP-1, a significant regression equation was found (F(6, 41) = 4.805, p < 0.001), with an R^2^ of 0.372. Participants’ predicted transformed TIMP-1 was equal to - 0.362 - 0.69 (COPD status) - 0.026 (sex) + 0.011 (age) + 0.083 (ICS usage) + 0.005 (pack-years) + 0.024 (smoking intensity). Smoking intensity was a significant predictor of transformed TIMP-1 levels in BAL.

*Interaction pQTM sensitivity analysis – the effect of DNAm outliers*

Since interaction analyses can be sensitive to outliers, we conducted a sensitivity analysis excluding potential DNA methylation outliers. This was done by applying an additional filtering step to remove DNA methylation values outside the range of (25th percentile - 3 * interquartile range [IQR]) and (75th percentile + 3 * IQR). After removing these values, the interaction pEWAS identified 59 significant interaction pQTMs, compared to the 66 identified in the original analysis. The majority of significant loci were consistent between the two analyses, with 55 loci overlapping.

**Supplemental table 1.** The top 10 Bonferroni-significant associations between BAL MMP-12 and DNA methylation in BAL cells (pQTMs), ranked by the magnitude of positive and negative effects (5 pQTMs each)

| CpG ID | Chr | Genomic Coordinate | Annotated to Gene | Coefficient | *p* value |
| --- | --- | --- | --- | --- | --- |
| cg12062537 | 1 | 1496856 | *SSU72** | 96.2 | 1.89 × 10^-8^ |
| cg02036387 | 11 | 65197533 | *FRMD8*† | 72.8 | 2.63 × 10^-8^ |
| cg14275457 | 9 | 132934675 | *NCS1** | 57.7 | 5.96 × 10^-8^ |
| cg10418263 | 6 | 35265544 | *DEF6** | 36.7 | 3.60 × 10^-8^ |
| cg22581895 | 3 | 30352425 | *TGFBR2*† | 22.8 | 2.25 × 10^-10^ |
| cg01625621 | 15 | 34260433 | *AVEN** | -32.1 | 1.36 × 10^-8^ |
| cg13436417 | 10 | 122262925 | *PLPP4* * | -24.9 | 1.85 × 10^-8^ |
| cg08777395 | 3 | 167682775 | *GOLIM4*† | -21.8 | 9.15 × 10^-10^ |
| cg21788523 | 8 | 3142662 | *CSMD1** | -21.8 | 1.19 × 10^-8^ |
| cg13193392 | 13 | 60055857 | *DIAPH3*† | -20.4 | 8.80 × 10^-10^ |

Definition of abbreviations: pQTM = protein quantitative trait methylation loci; MMP-12 = Matrix metallopeptidase 12; *SSU72* = SSU72 homolog, RNA polymerase II CTD phosphatase; *FRMD8* = FERM domain containing 8; *NCS1* = neuronal calcium sensor 1; *DEF6* = DEF6 guanine nucleotide exchange factor; *TGFBR2* = transforming growth factor beta receptor 2; *AVEN* = apoptosis and caspase activation inhibitor; *PLPP4* = Phospholipid Phosphatase 4: *GOLIM4* = golgi integral membrane protein 4; *CSMD1*= CUB and Sushi multiple domains 1; *DIAPH3* = diaphanous related formin 3. Adjusted for COPD, sex, age, ICS usage, cell type, pack-years, and smoking intensity. * Illumina annotation is based on the University of California, Santa Cruz (UCSC) database, using the hg19/GRCh37 genome assembly. † Nearest gene annotations are reported by the Genomic Regions Enrichment of Annotations Tool (GREAT), version 4.0.4, also based on the UCSC hg19/GRCh37 genome assembly.

**Supplemental figure 1.** The effect of COPD on the MMP-12–DNA methylation relationship – top 20 interaction pQTMs. X-axis: DNA methylation, y-axis: MMP-12 concentrations. MMP-12 = Matrix metalloproteinase 12; pQTM = protein quantitative trait methylation loci; COPD = chronic obstructive pulmonary disease.

**References**

1. Lindberg A, Linder R, Backman H, Ström JE, Frølich A, Nilsson U, et al. From COPD epidemiology to studies of pathophysiological disease mechanisms: challenges with regard to study design and recruitment process: Respiratory and Cardiovascular Effects in COPD (KOLIN). Eur Clin Respir J [Internet]. 2017;4:1415095. Available from: <https://www.tandfonline.com/doi/full/10.1080/20018525.2017.1415095>

2. Johnson WE, Li C, Rabinovic A. Adjusting batch effects in microarray expression data using empirical Bayes methods. Biostatistics [Internet]. 2007;8:118–27. Available from: <http://eutils.ncbi.nlm.nih.gov/entrez/eutils/elink.fcgi?dbfrom=pubmed&id=16632515&retmode=ref&cmd=prlinks>

3. Xu Z, Niu L, Li L, Taylor JA. ENmix: a novel background correction method for Illumina HumanMethylation450 BeadChip. Nucleic Acids Res [Internet]. 2016;44:e20. Available from: <http://eutils.ncbi.nlm.nih.gov/entrez/eutils/elink.fcgi?dbfrom=pubmed&id=26384415&retmode=ref&cmd=prlinks>

4. Zhou W, Laird PW, Shen H. Comprehensive characterization, annotation and innovative use of Infinium DNA methylation BeadChip probes. Nucleic Acids Res [Internet]. 2017;45:e22. Available from: <http://eutils.ncbi.nlm.nih.gov/entrez/eutils/elink.fcgi?dbfrom=pubmed&id=27924034&retmode=ref&cmd=prlinks>

5. Nordlund J, Bäcklin CL, Wahlberg P, Busche S, Berglund EC, Eloranta M-L, et al. Genome-wide signatures of differential DNA methylation in pediatric acute lymphoblastic leukemia. Genome Biol [Internet]. 2013;14:r105-15. Available from: <https://genomebiology.biomedcentral.com/articles/10.1186/gb-2013-14-9-r105>

6. Pidsley R, Wong CCY, Volta M, Lunnon K, Mill J, Schalkwyk LC. A data-driven approach to preprocessing Illumina 450K methylation array data. BMC Genomics [Internet]. 2013;14:293–10. Available from: <http://bmcgenomics.biomedcentral.com/articles/10.1186/1471-2164-14-293>

7. McLean CY, Bristor D, Hiller M, Clarke SL, Schaar BT, Lowe CB, et al. GREAT improves functional interpretation of cis-regulatory regions. Nature Publishing Group [Internet]. 2010;28:495–501. Available from: <http://eutils.ncbi.nlm.nih.gov/entrez/eutils/elink.fcgi?dbfrom=pubmed&id=20436461&retmode=ref&cmd=prlinks>
